# (*E*)-Methyl 2-[(2-formyl-6-meth­oxy­phen­oxy)meth­yl]-3-phenyl­acrylate

**DOI:** 10.1107/S1600536811054365

**Published:** 2011-12-23

**Authors:** T. Anuradha, G. Sivakumar, P. R. Seshadri, M. Bakthadoss

**Affiliations:** a Post Graduate & Research Department of Physics, Agurchand Manmull Jain College, Chennai 600 114, India; b Department of Organic Chemistry, University of Madras, Guindy Campus, Chennai 600 025, India

## Abstract

The title compound, C_19_H_18_O_5_, crystallizes with two independent mol­ecules (*A* and *B*) in an asymmetric unit in both of which the two aromatic rings are in a bis­ectional orientation as evidenced by the dihedral angle between them [41.7 (1)° in mol­ecule *A* and 35.6 (1)° in mol­ecule *B*]. Both mol­ecules adopt an *E* configuration with respect to the C=C bond. An intra­molecular C—H⋯O hydrogen-bond occurs in mol­ecule *A*. The crystal packing features inter­molecular C—H⋯O inter­actions.

## Related literature

For background to the synthesis, see: Bakthadoss *et al.* (2009 ▶). For related phenyl acrylate compounds, see: Wang *et al.* (2006 ▶); Jones & Jäger (2003 ▶). For their biological properties, see: Kim *et al.* (2004 ▶); Zhu *et al.* (2000 ▶).
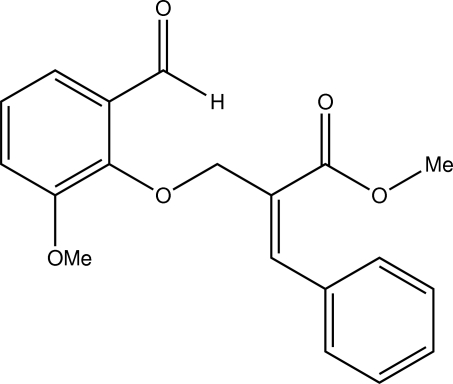


## Experimental

### 

#### Crystal data

C_19_H_18_O_5_
*M* *_r_* = 326.33
Triclinic, 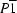

*a* = 8.4696 (5) Å
*b* = 12.1662 (7) Å
*c* = 16.9860 (9) Å
α = 94.423 (3)°
β = 100.038 (3)°
γ = 103.475 (3)°
*V* = 1663.33 (16) Å^3^
*Z* = 4
Mo *K*α radiation
μ = 0.09 mm^−1^
*T* = 293 K
0.20 × 0.20 × 0.20 mm

#### Data collection

Bruker SMART APEXII area-detector diffractometer
30114 measured reflections
8303 independent reflections
5258 reflections with *I* > 2σ(*I*)
*R*_int_ = 0.032

#### Refinement

*R*[*F* ^2^ > 2σ(*F* ^2^)] = 0.044
*wR*(*F* ^2^) = 0.137
*S* = 0.93
8303 reflections
433 parameters
H-atom parameters constrained
Δρ_max_ = 0.17 e Å^−3^
Δρ_min_ = −0.18 e Å^−3^

### 

Data collection: *APEX2* (Bruker, 2008 ▶); cell refinement: *SAINT* (Bruker, 2008 ▶); data reduction: *SAINT*; program(s) used to solve structure: *SHELXS97* (Sheldrick, 2008 ▶); program(s) used to refine structure: *SHELXL97* (Sheldrick, 2008 ▶); molecular graphics: *ORTEP-3* (Farrugia, 1997 ▶); software used to prepare material for publication: *SHELXL97*, *PLATON* (Spek, 2009 ▶) and *publCIF* (Westrip, 2010 ▶).

## Supplementary Material

Crystal structure: contains datablock(s) I, global. DOI: 10.1107/S1600536811054365/kp2375sup1.cif

Structure factors: contains datablock(s) I. DOI: 10.1107/S1600536811054365/kp2375Isup2.hkl

Supplementary material file. DOI: 10.1107/S1600536811054365/kp2375Isup3.cml

Additional supplementary materials:  crystallographic information; 3D view; checkCIF report

## Figures and Tables

**Table 1:** Hydrogen-bond geometry (Å, °)

*D*—H⋯*A*	*D*—H	H⋯*A*	*D*⋯*A*	*D*—H⋯*A*
C17*A*—H17*A*⋯O2*A*	0.93	2.48	3.354 (2)	156
C9*A*—H9*A*1⋯O4*B*^i^	0.97	2.55	3.2215 (16)	126
C19*B*—H19*D*⋯O5*A*^ii^	0.96	2.43	3.275 (3)	147

Symmetry codes: (i) 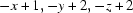; (ii) 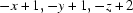.

**Fig. 1.**
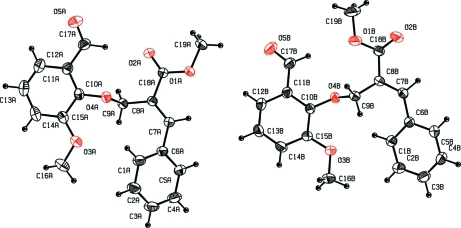
Molecular structure of the title compound, showing 30% probability displacement ellipsoids.

**Fig. 2.**
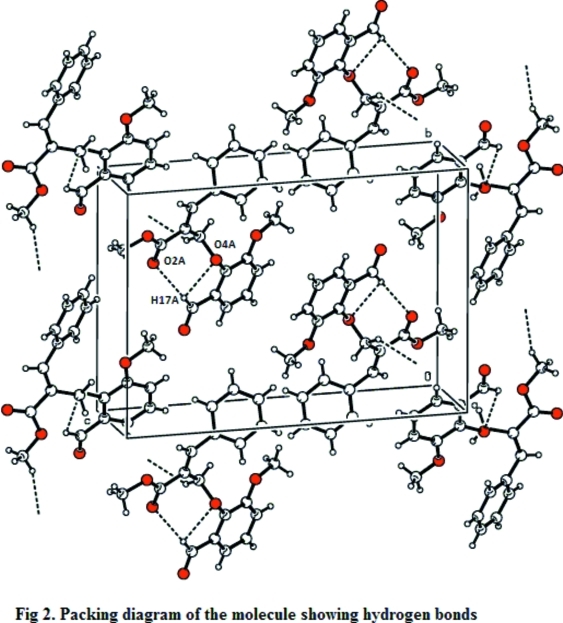
Packing of (I) with hydrogen bonds (dashed lines).

## supplementary crystallographic information

### Comment

Methyl cinnamate is used widely due to its flavour and fragrance in productions of cosmetics, beverages, baked goods, and convenience foods (Kim *et al.*, 2004). Cinnamic acid exhibits higher antifungal activity against *Asperigillus niger*, comparing to that of miconazole and a significant antifungal effect against *A. flavus* and *A. terreus*, while caffeic acid was inactive to the antifungal activity (Zhu *et al.*, 2000). In view of this medicinal importance, the crystal structure determination of the title compound was carried out and the results are presented here.

The asymmetric unit of the title compound contains the two independent molecules, A and B (Fig. 1). The dihedral angle between the C1 – C6 and C10 – C15 benzene rings is 41.7 (1)° in molecule A whereas it is 35.6 (1)° in molecule B. Both the molecules adopt *E* configurations with respect to the C ═ C bond. The methoxy and formyl group at the *meta* positions of the benzene group are close to being coplanar with the ring (5.7 (1)° and 4.2 (1)° in molecule A and 1.5 (1)° and 2.3 (1)° in molecule B). The central unit(C8/C9/C10/O4) is equatorial with respect to the phenylacrylate and formyl-methoxyphenyoxy (C1- C8/C18/C19/O1/O2 = 78.5 (1)° and C10 – C17/O3/O5 =69.5 (1)° in molecule A and 78.3 (1)° and 61.3 (2)° in molecule B). The acrylate group in molecule A is +*syn* periplanar with respect to central unit (C9/C8/C18/O2 = 2.7 (1)°) whereas in molecule B, the acrylate group is –antiperiplanar with respect to the central unit (C9/C8/C18/O2 = 176.7 (2)°) as evidenced by torsion angles.

The crystal packing is stabilised by intramolecular and intermolecular C — H··· O hydrogen bond interactions (Table 1, Fig. 2).

### Experimental

A solution of 2-hydroxy-3-methoxybenzaldehyde(1.0 mmol, 0.152 g) and potassium carbonate (2.0 mmol, 0.2293 g) in acetonitrile solvent (5 mL) was stirred for 15 m at room temperature. To this solution, (*Z*)-methyl-2-(bromomethyl)-3-phenylacrylate(1.2 mmol, 0.25 g) was added drop wise. After the completion of the reaction, as indicated by TLC, acetonitrile was evaporated. EtOAc (15 mL) and water (15 mL) were added to the crude mass. The organic layer was dried over anhydrous sodium sulfate. Removal of solvent led to the crude product, which was purified through pad of silica gel (100–200 mesh) using ethylacetate and hexanes (1:9) as solvents. The pure title compound was obtained as a colourless solid (0.31 g, 95% yield). Recrystallization was carried out using ethylacetate as a solvent.

### Refinement

All H atoms were positioned geometrically and allowed to ride on their parent atoms, with C—H = 0.93–0.97 Å and *U*_iso_(H) = 1.5*Ueq*(C) for methylH atoms and 1.2*U*eq(C) for other H atoms.

### Crystal data

**Table:**

C_19_H_18_O_5_	*Z* = 4
*M**_r_* = 326.33	*F*(000) = 688
Triclinic, *P*1	*D*_x_ = 1.303 Mg m^−^^3^
Hall symbol: -P 1	Mo *K*α radiation, λ = 0.71073 Å
*a* = 8.4696 (5) Å	Cell parameters from 8303 reflections
*b* = 12.1662 (7) Å	θ = 1.2–28.4°
*c* = 16.9860 (9) Å	µ = 0.09 mm^−^^1^
α = 94.423 (3)°	*T* = 293 K
β = 100.038 (3)°	Block, colourless
γ = 103.475 (3)°	0.20 × 0.20 × 0.20 mm
*V* = 1663.33 (16) Å^3^	

### Data collection

**Table:**

Bruker SMART APEXII area-detector diffractometer	5258 reflections with *I* > 2σ(*I*)
Radiation source: fine-focus sealed tube	*R*_int_ = 0.032
graphite	θ_max_ = 28.4°, θ_min_ = 1.2°
ω and φ scans	*h* = −11→11
30114 measured reflections	*k* = −16→16
8303 independent reflections	*l* = −21→22

### Refinement

**Table:**

Refinement on *F*^2^	Primary atom site location: structure-invariant direct methods
Least-squares matrix: full	Secondary atom site location: difference Fourier map
*R*[*F*^2^ > 2σ(*F*^2^)] = 0.044	Hydrogen site location: inferred from neighbouring sites
*wR*(*F*^2^) = 0.137	H-atom parameters constrained
*S* = 0.93	*w* = 1/[σ^2^(*F*_o_^2^) + (0.0725*P*)^2^ + 0.2552*P*] where *P* = (*F*_o_^2^ + 2*F*_c_^2^)/3
8303 reflections	(Δ/σ)_max_ = 0.001
433 parameters	Δρ_max_ = 0.17 e Å^−^^3^
0 restraints	Δρ_min_ = −0.18 e Å^−^^3^

### Special details

**Table:**

Geometry. All e.s.d.'s (except the e.s.d. in the dihedral angle between two l.s. planes) are estimated using the full covariance matrix. The cell e.s.d.'s are taken into account individually in the estimation of e.s.d.'s in distances, angles and torsion angles; correlations between e.s.d.'s in cell parameters are only used when they are defined by crystal symmetry. An approximate (isotropic) treatment of cell e.s.d.'s is used for estimating e.s.d.'s involving l.s. planes.
Refinement. Refinement of *F*^2^ against ALL reflections. The weighted *R*-factor *wR* and goodness of fit *S* are based on *F*^2^, conventional *R*-factors *R* are based on *F*, with *F* set to zero for negative *F*^2^. The threshold expression of *F*^2^ > σ(*F*^2^) is used only for calculating *R*-factors(gt) *etc*. and is not relevant to the choice of reflections for refinement. *R*-factors based on *F*^2^ are statistically about twice as large as those based on *F*, and *R*- factors based on ALL data will be even larger.

### Fractional atomic coordinates and isotropic or equivalent isotropic displacement parameters (Å^2^)

**Table:**

	*x*	*y*	*z*	*U*_iso_*/*U*_eq_	
O1A	0.41536 (13)	0.75129 (9)	0.88919 (6)	0.0567 (3)	
O2A	0.18473 (14)	0.61117 (9)	0.85240 (7)	0.0611 (3)	
O3A	−0.05209 (15)	0.66404 (10)	0.52848 (7)	0.0641 (3)	
O4A	0.10199 (11)	0.59456 (8)	0.65973 (6)	0.0449 (2)	
O5A	−0.0729 (2)	0.29588 (11)	0.73737 (9)	0.0909 (5)	
C1A	0.2947 (2)	0.85775 (14)	0.60506 (10)	0.0600 (4)	
H1A	0.2651	0.7787	0.5951	0.072*	
C2A	0.2924 (3)	0.92180 (17)	0.54131 (11)	0.0724 (5)	
H2A	0.2621	0.8855	0.4887	0.087*	
C3A	0.3346 (3)	1.03855 (17)	0.55494 (12)	0.0740 (5)	
H3A	0.3322	1.0812	0.5118	0.089*	
C4A	0.3800 (3)	1.09178 (16)	0.63207 (12)	0.0733 (5)	
H4A	0.4077	1.1709	0.6414	0.088*	
C5A	0.3851 (2)	1.02901 (14)	0.69644 (10)	0.0630 (4)	
H5A	0.4185	1.0661	0.7488	0.076*	
C6A	0.34062 (19)	0.91055 (13)	0.68349 (9)	0.0490 (3)	
C7A	0.34918 (18)	0.84624 (12)	0.75324 (9)	0.0473 (3)	
H7A	0.4384	0.8765	0.7957	0.057*	
C8A	0.24490 (17)	0.74965 (12)	0.76346 (8)	0.0417 (3)	
C9A	0.08546 (17)	0.69396 (12)	0.70716 (8)	0.0432 (3)	
H9A1	−0.0012	0.6715	0.7376	0.052*	
H9A2	0.0543	0.7474	0.6717	0.052*	
C10A	−0.04856 (17)	0.52761 (12)	0.61780 (8)	0.0455 (3)	
C11A	−0.11578 (19)	0.42426 (13)	0.64278 (9)	0.0512 (4)	
C12A	−0.2642 (2)	0.35353 (15)	0.59765 (12)	0.0664 (5)	
H12A	−0.3094	0.2836	0.6138	0.080*	
C13A	−0.3422 (2)	0.38740 (18)	0.53019 (12)	0.0732 (5)	
H13A	−0.4405	0.3402	0.5003	0.088*	
C14A	−0.2767 (2)	0.49130 (17)	0.50553 (10)	0.0660 (5)	
H14A	−0.3322	0.5136	0.4597	0.079*	
C15A	−0.12979 (19)	0.56217 (14)	0.54836 (9)	0.0523 (4)	
C16A	−0.1390 (3)	0.71142 (19)	0.46640 (11)	0.0779 (6)	
H16A	−0.0706	0.7830	0.4586	0.117*	
H16B	−0.2387	0.7232	0.4813	0.117*	
H16C	−0.1667	0.6602	0.4173	0.117*	
C17A	−0.0340 (2)	0.38920 (14)	0.71684 (11)	0.0613 (4)	
H17A	0.0537	0.4427	0.7500	0.074*	
C18A	0.27580 (17)	0.69571 (12)	0.83820 (8)	0.0436 (3)	
C19A	0.4474 (2)	0.70544 (17)	0.96434 (10)	0.0729 (5)	
H19A	0.5496	0.7507	0.9968	0.109*	
H19B	0.4553	0.6284	0.9539	0.109*	
H19C	0.3586	0.7067	0.9924	0.109*	
O1B	1.29774 (17)	1.18987 (11)	1.37809 (7)	0.0731 (4)	
O2B	1.1832 (2)	1.06673 (10)	1.26819 (7)	0.0812 (4)	
O3B	1.12347 (15)	1.32548 (10)	1.01612 (7)	0.0654 (3)	
O4B	1.04928 (12)	1.21083 (8)	1.14210 (5)	0.0453 (2)	
O5B	0.7023 (2)	0.91721 (12)	1.11274 (8)	0.0979 (5)	
C1B	1.2973 (2)	1.49121 (13)	1.18301 (9)	0.0533 (4)	
H1B	1.1923	1.4422	1.1666	0.064*	
C2B	1.3363 (2)	1.59176 (14)	1.15034 (11)	0.0637 (4)	
H2B	1.2567	1.6106	1.1125	0.076*	
C3B	1.4919 (2)	1.66485 (15)	1.17302 (11)	0.0667 (5)	
H3B	1.5172	1.7326	1.1506	0.080*	
C4B	1.6089 (2)	1.63690 (15)	1.22894 (11)	0.0685 (5)	
H4B	1.7145	1.6854	1.2440	0.082*	
C5B	1.5709 (2)	1.53742 (14)	1.26295 (10)	0.0587 (4)	
H5B	1.6509	1.5200	1.3014	0.070*	
C6B	1.41469 (18)	1.46261 (12)	1.24054 (8)	0.0460 (3)	
C7B	1.37621 (18)	1.36008 (13)	1.28042 (9)	0.0473 (3)	
H7B	1.4189	1.3692	1.3355	0.057*	
C8B	1.28832 (17)	1.25531 (12)	1.24876 (8)	0.0435 (3)	
C9B	1.22565 (18)	1.21970 (13)	1.16011 (8)	0.0447 (3)	
H9B1	1.2822	1.2754	1.1299	0.054*	
H9B2	1.2470	1.1468	1.1449	0.054*	
C10B	0.96683 (17)	1.16893 (12)	1.06435 (8)	0.0426 (3)	
C11B	0.84002 (18)	1.06988 (12)	1.05216 (9)	0.0476 (3)	
C12B	0.7411 (2)	1.03311 (14)	0.97533 (10)	0.0594 (4)	
H12B	0.6534	0.9685	0.9673	0.071*	
C13B	0.7728 (2)	1.09160 (15)	0.91246 (10)	0.0632 (4)	
H13B	0.7069	1.0662	0.8616	0.076*	
C14B	0.9019 (2)	1.18846 (14)	0.92324 (9)	0.0577 (4)	
H14B	0.9239	1.2266	0.8794	0.069*	
C15B	0.99822 (19)	1.22864 (12)	0.99897 (9)	0.0484 (3)	
C16B	1.1582 (3)	1.38958 (19)	0.95210 (12)	0.0898 (7)	
H16D	1.2485	1.4552	0.9723	0.135*	
H16E	1.0619	1.4136	0.9291	0.135*	
H16F	1.1879	1.3433	0.9115	0.135*	
C17B	0.8112 (2)	1.00228 (14)	1.11870 (10)	0.0616 (4)	
H17B	0.8818	1.0270	1.1685	0.074*	
C18B	1.2594 (2)	1.16990 (13)	1.30624 (9)	0.0508 (4)	
C19B	1.1463 (4)	0.97776 (18)	1.31915 (15)	0.1193 (10)	
H19D	1.0918	0.9068	1.2861	0.179*	
H19E	1.0752	0.9967	1.3535	0.179*	
H19F	1.2475	0.9708	1.3516	0.179*	

### Atomic displacement parameters (Å^2^)

**Table:**

	*U*^11^	*U*^22^	*U*^33^	*U*^12^	*U*^13^	*U*^23^
O1A	0.0527 (6)	0.0632 (7)	0.0429 (6)	−0.0024 (5)	−0.0035 (5)	0.0188 (5)
O2A	0.0648 (7)	0.0522 (6)	0.0551 (6)	−0.0041 (5)	0.0021 (5)	0.0186 (5)
O3A	0.0669 (7)	0.0720 (8)	0.0502 (6)	0.0178 (6)	−0.0018 (5)	0.0172 (6)
O4A	0.0418 (5)	0.0463 (5)	0.0446 (5)	0.0123 (4)	0.0035 (4)	0.0019 (4)
O5A	0.1161 (12)	0.0530 (8)	0.0974 (10)	0.0087 (7)	0.0142 (9)	0.0254 (7)
C1A	0.0791 (11)	0.0507 (9)	0.0529 (9)	0.0140 (8)	0.0204 (8)	0.0130 (7)
C2A	0.1036 (15)	0.0701 (12)	0.0463 (9)	0.0214 (11)	0.0194 (9)	0.0151 (8)
C3A	0.1022 (15)	0.0667 (12)	0.0617 (11)	0.0232 (11)	0.0262 (10)	0.0313 (9)
C4A	0.0986 (14)	0.0498 (10)	0.0734 (12)	0.0122 (9)	0.0245 (11)	0.0219 (9)
C5A	0.0776 (12)	0.0507 (9)	0.0545 (10)	0.0029 (8)	0.0117 (8)	0.0118 (7)
C6A	0.0506 (8)	0.0481 (8)	0.0485 (8)	0.0096 (7)	0.0106 (7)	0.0149 (7)
C7A	0.0479 (8)	0.0472 (8)	0.0441 (8)	0.0081 (6)	0.0054 (6)	0.0104 (6)
C8A	0.0442 (7)	0.0427 (7)	0.0391 (7)	0.0130 (6)	0.0067 (6)	0.0074 (6)
C9A	0.0438 (7)	0.0449 (8)	0.0411 (7)	0.0132 (6)	0.0061 (6)	0.0054 (6)
C10A	0.0431 (8)	0.0487 (8)	0.0427 (8)	0.0122 (6)	0.0059 (6)	−0.0020 (6)
C11A	0.0519 (9)	0.0473 (8)	0.0529 (9)	0.0099 (7)	0.0128 (7)	−0.0013 (7)
C12A	0.0603 (10)	0.0566 (10)	0.0731 (12)	0.0009 (8)	0.0139 (9)	−0.0068 (9)
C13A	0.0512 (10)	0.0788 (13)	0.0739 (13)	0.0029 (9)	0.0000 (9)	−0.0163 (10)
C14A	0.0551 (10)	0.0851 (13)	0.0510 (10)	0.0198 (9)	−0.0040 (8)	−0.0066 (9)
C15A	0.0522 (9)	0.0604 (10)	0.0436 (8)	0.0167 (7)	0.0061 (7)	0.0009 (7)
C16A	0.0868 (14)	0.0971 (15)	0.0559 (11)	0.0384 (12)	0.0037 (9)	0.0244 (10)
C17A	0.0712 (11)	0.0468 (9)	0.0646 (10)	0.0111 (8)	0.0141 (8)	0.0083 (8)
C18A	0.0457 (8)	0.0426 (8)	0.0414 (7)	0.0092 (6)	0.0074 (6)	0.0064 (6)
C19A	0.0722 (11)	0.0837 (13)	0.0488 (10)	0.0002 (10)	−0.0074 (8)	0.0276 (9)
O1B	0.1041 (10)	0.0693 (8)	0.0423 (7)	0.0151 (7)	0.0101 (6)	0.0163 (6)
O2B	0.1318 (12)	0.0456 (7)	0.0557 (7)	0.0084 (7)	0.0048 (7)	0.0166 (6)
O3B	0.0734 (8)	0.0593 (7)	0.0506 (6)	−0.0068 (6)	0.0032 (5)	0.0218 (5)
O4B	0.0458 (5)	0.0503 (6)	0.0354 (5)	0.0058 (4)	0.0065 (4)	0.0019 (4)
O5B	0.1163 (12)	0.0733 (9)	0.0750 (9)	−0.0361 (9)	0.0218 (8)	0.0103 (7)
C1B	0.0493 (8)	0.0524 (9)	0.0559 (9)	0.0090 (7)	0.0073 (7)	0.0111 (7)
C2B	0.0691 (11)	0.0580 (10)	0.0648 (11)	0.0171 (9)	0.0088 (8)	0.0191 (8)
C3B	0.0824 (13)	0.0489 (9)	0.0659 (11)	0.0053 (9)	0.0204 (10)	0.0124 (8)
C4B	0.0652 (11)	0.0587 (10)	0.0677 (11)	−0.0079 (8)	0.0091 (9)	0.0045 (9)
C5B	0.0558 (9)	0.0584 (10)	0.0522 (9)	0.0038 (8)	0.0000 (7)	0.0042 (7)
C6B	0.0498 (8)	0.0465 (8)	0.0391 (7)	0.0085 (6)	0.0078 (6)	0.0030 (6)
C7B	0.0476 (8)	0.0534 (9)	0.0385 (7)	0.0113 (7)	0.0031 (6)	0.0067 (6)
C8B	0.0459 (8)	0.0479 (8)	0.0391 (7)	0.0161 (6)	0.0069 (6)	0.0094 (6)
C9B	0.0483 (8)	0.0472 (8)	0.0402 (7)	0.0143 (6)	0.0090 (6)	0.0073 (6)
C10B	0.0460 (8)	0.0438 (8)	0.0370 (7)	0.0107 (6)	0.0069 (6)	0.0023 (6)
C11B	0.0524 (8)	0.0428 (8)	0.0442 (8)	0.0069 (6)	0.0101 (6)	0.0000 (6)
C12B	0.0619 (10)	0.0513 (9)	0.0534 (9)	0.0012 (8)	0.0027 (8)	−0.0037 (7)
C13B	0.0735 (11)	0.0591 (10)	0.0464 (9)	0.0120 (9)	−0.0062 (8)	−0.0026 (8)
C14B	0.0725 (11)	0.0590 (10)	0.0410 (8)	0.0179 (8)	0.0052 (7)	0.0110 (7)
C15B	0.0550 (9)	0.0439 (8)	0.0443 (8)	0.0094 (7)	0.0074 (7)	0.0091 (6)
C16B	0.0915 (15)	0.0893 (15)	0.0724 (13)	−0.0126 (12)	0.0048 (11)	0.0455 (11)
C17B	0.0712 (11)	0.0522 (9)	0.0531 (9)	−0.0027 (8)	0.0161 (8)	0.0046 (7)
C18B	0.0608 (9)	0.0489 (9)	0.0441 (9)	0.0178 (7)	0.0068 (7)	0.0106 (7)
C19B	0.199 (3)	0.0553 (12)	0.0878 (16)	0.0041 (15)	0.0135 (17)	0.0348 (11)

### Geometric parameters (Å, °)

**Table:**

O1A—C18A	1.3360 (17)	O1B—C18B	1.1971 (18)
O1A—C19A	1.4400 (18)	O2B—C18B	1.327 (2)
O2A—C18A	1.2019 (17)	O2B—C19B	1.449 (2)
O3A—C15A	1.358 (2)	O3B—C15B	1.3620 (18)
O3A—C16A	1.417 (2)	O3B—C16B	1.419 (2)
O4A—C10A	1.3808 (17)	O4B—C10B	1.3785 (16)
O4A—C9A	1.4488 (17)	O4B—C9B	1.4481 (17)
O5A—C17A	1.202 (2)	O5B—C17B	1.1993 (19)
C1A—C6A	1.382 (2)	C1B—C2B	1.376 (2)
C1A—C2A	1.382 (2)	C1B—C6B	1.393 (2)
C1A—H1A	0.9300	C1B—H1B	0.9300
C2A—C3A	1.372 (3)	C2B—C3B	1.378 (3)
C2A—H2A	0.9300	C2B—H2B	0.9300
C3A—C4A	1.364 (3)	C3B—C4B	1.371 (3)
C3A—H3A	0.9300	C3B—H3B	0.9300
C4A—C5A	1.381 (2)	C4B—C5B	1.376 (2)
C4A—H4A	0.9300	C4B—H4B	0.9300
C5A—C6A	1.392 (2)	C5B—C6B	1.391 (2)
C5A—H5A	0.9300	C5B—H5B	0.9300
C6A—C7A	1.470 (2)	C6B—C7B	1.463 (2)
C7A—C8A	1.339 (2)	C7B—C8B	1.334 (2)
C7A—H7A	0.9300	C7B—H7B	0.9300
C8A—C18A	1.4844 (19)	C8B—C18B	1.488 (2)
C8A—C9A	1.4902 (19)	C8B—C9B	1.5002 (19)
C9A—H9A1	0.9700	C9B—H9B1	0.9700
C9A—H9A2	0.9700	C9B—H9B2	0.9700
C10A—C11A	1.385 (2)	C10B—C11B	1.389 (2)
C10A—C15A	1.403 (2)	C10B—C15B	1.403 (2)
C11A—C12A	1.403 (2)	C11B—C12B	1.400 (2)
C11A—C17A	1.470 (2)	C11B—C17B	1.469 (2)
C12A—C13A	1.362 (3)	C12B—C13B	1.359 (2)
C12A—H12A	0.9300	C12B—H12B	0.9300
C13A—C14A	1.384 (3)	C13B—C14B	1.383 (2)
C13A—H13A	0.9300	C13B—H13B	0.9300
C14A—C15A	1.382 (2)	C14B—C15B	1.382 (2)
C14A—H14A	0.9300	C14B—H14B	0.9300
C16A—H16A	0.9600	C16B—H16D	0.9600
C16A—H16B	0.9600	C16B—H16E	0.9600
C16A—H16C	0.9600	C16B—H16F	0.9600
C17A—H17A	0.9300	C17B—H17B	0.9300
C19A—H19A	0.9600	C19B—H19D	0.9600
C19A—H19B	0.9600	C19B—H19E	0.9600
C19A—H19C	0.9600	C19B—H19F	0.9600
			
C18A—O1A—C19A	115.45 (12)	C18B—O2B—C19B	115.86 (15)
C15A—O3A—C16A	118.01 (14)	C15B—O3B—C16B	118.03 (13)
C10A—O4A—C9A	112.60 (10)	C10B—O4B—C9B	116.89 (10)
C6A—C1A—C2A	120.38 (16)	C2B—C1B—C6B	120.19 (15)
C6A—C1A—H1A	119.8	C2B—C1B—H1B	119.9
C2A—C1A—H1A	119.8	C6B—C1B—H1B	119.9
C3A—C2A—C1A	120.54 (17)	C1B—C2B—C3B	120.84 (17)
C3A—C2A—H2A	119.7	C1B—C2B—H2B	119.6
C1A—C2A—H2A	119.7	C3B—C2B—H2B	119.6
C4A—C3A—C2A	119.68 (17)	C4B—C3B—C2B	119.44 (17)
C4A—C3A—H3A	120.2	C4B—C3B—H3B	120.3
C2A—C3A—H3A	120.2	C2B—C3B—H3B	120.3
C3A—C4A—C5A	120.48 (17)	C3B—C4B—C5B	120.37 (17)
C3A—C4A—H4A	119.8	C3B—C4B—H4B	119.8
C5A—C4A—H4A	119.8	C5B—C4B—H4B	119.8
C4A—C5A—C6A	120.41 (17)	C4B—C5B—C6B	120.89 (16)
C4A—C5A—H5A	119.8	C4B—C5B—H5B	119.6
C6A—C5A—H5A	119.8	C6B—C5B—H5B	119.6
C1A—C6A—C5A	118.48 (14)	C5B—C6B—C1B	118.27 (14)
C1A—C6A—C7A	122.41 (14)	C5B—C6B—C7B	119.47 (14)
C5A—C6A—C7A	119.08 (14)	C1B—C6B—C7B	122.19 (13)
C8A—C7A—C6A	128.24 (14)	C8B—C7B—C6B	128.96 (13)
C8A—C7A—H7A	115.9	C8B—C7B—H7B	115.5
C6A—C7A—H7A	115.9	C6B—C7B—H7B	115.5
C7A—C8A—C18A	120.49 (13)	C7B—C8B—C18B	116.47 (13)
C7A—C8A—C9A	124.49 (13)	C7B—C8B—C9B	123.79 (13)
C18A—C8A—C9A	114.79 (12)	C18B—C8B—C9B	119.68 (13)
O4A—C9A—C8A	110.18 (11)	O4B—C9B—C8B	108.60 (11)
O4A—C9A—H9A1	109.6	O4B—C9B—H9B1	110.0
C8A—C9A—H9A1	109.6	C8B—C9B—H9B1	110.0
O4A—C9A—H9A2	109.6	O4B—C9B—H9B2	110.0
C8A—C9A—H9A2	109.6	C8B—C9B—H9B2	110.0
H9A1—C9A—H9A2	108.1	H9B1—C9B—H9B2	108.4
O4A—C10A—C11A	119.70 (13)	O4B—C10B—C11B	118.40 (12)
O4A—C10A—C15A	120.06 (13)	O4B—C10B—C15B	121.71 (13)
C11A—C10A—C15A	120.19 (14)	C11B—C10B—C15B	119.66 (13)
C10A—C11A—C12A	119.64 (16)	C10B—C11B—C12B	119.55 (14)
C10A—C11A—C17A	120.45 (14)	C10B—C11B—C17B	120.62 (13)
C12A—C11A—C17A	119.90 (15)	C12B—C11B—C17B	119.82 (14)
C13A—C12A—C11A	119.87 (17)	C13B—C12B—C11B	120.21 (15)
C13A—C12A—H12A	120.1	C13B—C12B—H12B	119.9
C11A—C12A—H12A	120.1	C11B—C12B—H12B	119.9
C12A—C13A—C14A	120.74 (17)	C12B—C13B—C14B	120.83 (15)
C12A—C13A—H13A	119.6	C12B—C13B—H13B	119.6
C14A—C13A—H13A	119.6	C14B—C13B—H13B	119.6
C15A—C14A—C13A	120.63 (17)	C15B—C14B—C13B	120.14 (15)
C15A—C14A—H14A	119.7	C15B—C14B—H14B	119.9
C13A—C14A—H14A	119.7	C13B—C14B—H14B	119.9
O3A—C15A—C14A	125.62 (15)	O3B—C15B—C14B	124.42 (14)
O3A—C15A—C10A	115.46 (13)	O3B—C15B—C10B	116.01 (13)
C14A—C15A—C10A	118.91 (16)	C14B—C15B—C10B	119.55 (14)
O3A—C16A—H16A	109.5	O3B—C16B—H16D	109.5
O3A—C16A—H16B	109.5	O3B—C16B—H16E	109.5
H16A—C16A—H16B	109.5	H16D—C16B—H16E	109.5
O3A—C16A—H16C	109.5	O3B—C16B—H16F	109.5
H16A—C16A—H16C	109.5	H16D—C16B—H16F	109.5
H16B—C16A—H16C	109.5	H16E—C16B—H16F	109.5
O5A—C17A—C11A	124.33 (17)	O5B—C17B—C11B	124.08 (16)
O5A—C17A—H17A	117.8	O5B—C17B—H17B	118.0
C11A—C17A—H17A	117.8	C11B—C17B—H17B	118.0
O2A—C18A—O1A	122.83 (13)	O1B—C18B—O2B	123.09 (15)
O2A—C18A—C8A	123.97 (13)	O1B—C18B—C8B	125.27 (15)
O1A—C18A—C8A	113.20 (12)	O2B—C18B—C8B	111.64 (13)
O1A—C19A—H19A	109.5	O2B—C19B—H19D	109.5
O1A—C19A—H19B	109.5	O2B—C19B—H19E	109.5
H19A—C19A—H19B	109.5	H19D—C19B—H19E	109.5
O1A—C19A—H19C	109.5	O2B—C19B—H19F	109.5
H19A—C19A—H19C	109.5	H19D—C19B—H19F	109.5
H19B—C19A—H19C	109.5	H19E—C19B—H19F	109.5
			
C6A—C1A—C2A—C3A	−0.5 (3)	C6B—C1B—C2B—C3B	0.9 (3)
C1A—C2A—C3A—C4A	0.4 (3)	C1B—C2B—C3B—C4B	−0.1 (3)
C2A—C3A—C4A—C5A	0.5 (3)	C2B—C3B—C4B—C5B	−0.8 (3)
C3A—C4A—C5A—C6A	−1.4 (3)	C3B—C4B—C5B—C6B	1.0 (3)
C2A—C1A—C6A—C5A	−0.4 (3)	C4B—C5B—C6B—C1B	−0.3 (2)
C2A—C1A—C6A—C7A	−178.36 (16)	C4B—C5B—C6B—C7B	−177.33 (15)
C4A—C5A—C6A—C1A	1.3 (3)	C2B—C1B—C6B—C5B	−0.7 (2)
C4A—C5A—C6A—C7A	179.34 (16)	C2B—C1B—C6B—C7B	176.33 (15)
C1A—C6A—C7A—C8A	−40.2 (2)	C5B—C6B—C7B—C8B	−141.00 (17)
C5A—C6A—C7A—C8A	141.79 (17)	C1B—C6B—C7B—C8B	42.0 (2)
C6A—C7A—C8A—C18A	178.94 (14)	C6B—C7B—C8B—C18B	−175.41 (14)
C6A—C7A—C8A—C9A	−6.8 (2)	C6B—C7B—C8B—C9B	7.3 (2)
C10A—O4A—C9A—C8A	168.47 (11)	C10B—O4B—C9B—C8B	−174.31 (11)
C7A—C8A—C9A—O4A	104.79 (16)	C7B—C8B—C9B—O4B	−105.02 (16)
C18A—C8A—C9A—O4A	−80.66 (14)	C18B—C8B—C9B—O4B	77.76 (16)
C9A—O4A—C10A—C11A	−106.93 (14)	C9B—O4B—C10B—C11B	119.51 (14)
C9A—O4A—C10A—C15A	75.55 (16)	C9B—O4B—C10B—C15B	−66.03 (18)
O4A—C10A—C11A—C12A	−176.58 (13)	O4B—C10B—C11B—C12B	172.24 (14)
C15A—C10A—C11A—C12A	0.9 (2)	C15B—C10B—C11B—C12B	−2.3 (2)
O4A—C10A—C11A—C17A	4.4 (2)	O4B—C10B—C11B—C17B	−9.1 (2)
C15A—C10A—C11A—C17A	−178.09 (14)	C15B—C10B—C11B—C17B	176.36 (14)
C10A—C11A—C12A—C13A	−0.7 (3)	C10B—C11B—C12B—C13B	2.4 (2)
C17A—C11A—C12A—C13A	178.30 (17)	C17B—C11B—C12B—C13B	−176.30 (16)
C11A—C12A—C13A—C14A	−0.1 (3)	C11B—C12B—C13B—C14B	−0.5 (3)
C12A—C13A—C14A—C15A	0.8 (3)	C12B—C13B—C14B—C15B	−1.6 (3)
C16A—O3A—C15A—C14A	10.6 (2)	C16B—O3B—C15B—C14B	−0.2 (3)
C16A—O3A—C15A—C10A	−170.80 (15)	C16B—O3B—C15B—C10B	−179.14 (17)
C13A—C14A—C15A—O3A	177.99 (16)	C13B—C14B—C15B—O3B	−177.30 (16)
C13A—C14A—C15A—C10A	−0.6 (2)	C13B—C14B—C15B—C10B	1.6 (2)
O4A—C10A—C15A—O3A	−1.5 (2)	O4B—C10B—C15B—O3B	4.9 (2)
C11A—C10A—C15A—O3A	−179.01 (13)	C11B—C10B—C15B—O3B	179.35 (13)
O4A—C10A—C15A—C14A	177.22 (14)	O4B—C10B—C15B—C14B	−174.06 (14)
C11A—C10A—C15A—C14A	−0.3 (2)	C11B—C10B—C15B—C14B	0.3 (2)
C10A—C11A—C17A—O5A	−170.69 (17)	C10B—C11B—C17B—O5B	178.61 (18)
C12A—C11A—C17A—O5A	10.3 (3)	C12B—C11B—C17B—O5B	−2.7 (3)
C19A—O1A—C18A—O2A	−2.1 (2)	C19B—O2B—C18B—O1B	0.6 (3)
C19A—O1A—C18A—C8A	177.28 (14)	C19B—O2B—C18B—C8B	−178.78 (19)
C7A—C8A—C18A—O2A	177.55 (15)	C7B—C8B—C18B—O1B	5.9 (2)
C9A—C8A—C18A—O2A	2.8 (2)	C9B—C8B—C18B—O1B	−176.72 (15)
C7A—C8A—C18A—O1A	−1.85 (19)	C7B—C8B—C18B—O2B	−174.76 (15)
C9A—C8A—C18A—O1A	−176.63 (12)	C9B—C8B—C18B—O2B	2.7 (2)

### Hydrogen-bond geometry (Å, °)

**Table:**

*D*—H···*A*	*D*—H	H···*A*	*D*···*A*	*D*—H···*A*
C17A—H17A···O2A	0.93	2.48	3.354 (2)	156.
C17A—H17A···O4A	0.93	2.50	2.8164 (19)	100.
C17B—H17B···O4B	0.93	2.47	2.8004 (19)	101.
C9A—H9A2···O3A	0.97	2.49	3.0167 (18)	114.
C9B—H9B1···O3B	0.97	2.36	2.9416 (17)	118.
C7B—H7B···O1B	0.93	2.41	2.7847 (18)	104.
C7A—H7A···O1A	0.93	2.29	2.6914 (17)	106.
C9A—H9A1···O4B^i^	0.97	2.55	3.2215 (16)	126.
C19B—H19D···O5A^ii^	0.96	2.43	3.275 (3)	147.

Symmetry codes: (i) −*x*+1, −*y*+2, −*z*+2; (ii) −*x*+1, −*y*+1, −*z*+2.
